# An ROR1 bi-specific T-cell engager provides effective targeting and cytotoxicity against a range of solid tumors

**DOI:** 10.1080/2162402X.2017.1326437

**Published:** 2017-05-17

**Authors:** Satyen Harish Gohil, Solange Rosa Paredes-Moscosso, Micaela Harrasser, Marzia Vezzalini, Aldo Scarpa, Emma Morris, Andrew M. Davidoff, Claudio Sorio, Amit Chunilal Nathwani, Marco Della Peruta

**Affiliations:** a Department of Academic Haematology, University College London Cancer Institute, London, UK; b Katharine Dormandy Haemophilia and Thrombosis Centre, London, UK; c National Health Service Blood and Transplant, Oak House, Reeds Crescent, Watford, Hertfordshire, UK; d Department of Pathology and Diagnostics, University of Verona Medical School, Verona, Italy; e Institute of Immunity and Transplantation, University College London, Royal Free Hospital, Pond Street, London, UK; f Department of Surgery, St. Jude Children's Research Hospital, Memphis, TN, USA

**Keywords:** Cancer, Immunotherapy, ROR1

## Abstract

We have developed a humanized bi-specific T-cell engager (BiTE) targeting receptor tyrosine kinase-like orphan receptor 1 (ROR1), a cell surface antigen present on a range of malignancies and cancer-initiating cells. Focusing initially on pancreatic cancer, we demonstrated that our ROR1 BiTE results in T cell mediated and antigen-specific cytotoxicity against ROR1-expressing pancreatic cancer cell lines *in vitro* at exceedingly low concentrations (0.1 ng/mL) and low effector to target ratios. Our BiTE prevented engraftment of pancreatic tumor xenografts in murine models and reduced the size of established subcutaneous tumors by at least 3-fold. To validate its wider therapeutic potential, we next demonstrated significant cytotoxicity against ovarian cancer in an *in vitro* and *in vivo* setting and T-cell-mediated killing of a range of histologically distinct solid tumor cell lines. Overall, our ROR1 BiTE represents a promising immunotherapy approach, because of its ability to target a broad range of malignancies, many with significant unmet therapeutic needs.

## Introduction

Currently, over 8 million people die of cancer annually worldwide^1^ and although cancer survival has doubled over the last 40 y, for some tumors such as pancreatic cancer the outlooks remain dismal. Attention has therefore turned to immunotherapy, which has achieved notable success in the treatment of malignancies over the last decade. Monoclonal antibodies targeting antigens expressed on tumors have improved survival in patients with B cell lymphoid malignancies and breast cancer.^2^^,^^3^ Antibodies that activate endogenous T cells by blocking immune checkpoints are beginning to generate durable clinical responses in patients with melanoma, lung, renal, and bladder cancers but appear to be less effective in certain tumors such as pancreatic carcinoma.^4-6^ Adoptive T cell immunotherapy with genetically modified T cells that express chimeric antigen receptors (CARs) show great promise in the treatment of hematological malignancies, especially in patients with refractory B-cell acute lymphoblastic leukemia (B-ALL).^7^^,^^8^ These artificial receptors, consisting of a tumor-targeting single-chain variable fragment linked to one or more intracellular T cell receptor (TCR) signaling domains, enable targeting of tumor antigens in an major histocompatibility complex (MHC) unrestricted manner.

Bi-specific T-cell engagers (BiTEs) provide an alternative to CAR T-cells as they dispense with the need for *ex vivo* manipulation and engineering of T cells.^9^ BiTEs consist of small flexible molecules composed of two antibody-derived single chain variable fragments (scFv) linked in tandem. One arm targets the TCR CD3 subunit, while the second binds to a tumor-associated antigen (e.g., CD19). BiTEs can redirect endogenous polyclonal T cells to sites of tumors where, upon engagement with tumor antigen, they promote the formation of immunological synapses. This is followed by the release of perforins, granzyme B, and cytokines, and selective killing of tumor cells independently of MHC, costimulatory molecules, and antigen presentation.^9^^,^^10^ Blinatumomab, the first in class BiTE, targets CD19 and is highly effective in the treatment of chemotherapy-resistant relapsed/refractory B-ALL patients.^11-13^ As CD19 is exclusively expressed on B-lymphocytes, Blinatumomab cannot be used for the treatment of other cancers with significant unmet need, such as pancreatic cancer. Therefore, BiTEs with broad applicability across a range of cancer types are required.

Receptor tyrosine kinase-like orphan receptor 1 (ROR1) is a surface antigen present at high levels on an array of hematological malignancies and solid tumors, including pancreatic,^14^^,^^15^ ovarian,^14-18^ breast,^14^^,^^19-21^ lung,^14^^,^^22^^,^^23^ and gastric cancer^24^ as well as melanoma,^25^^,^^26^ Ewing sarcoma,^27^ chronic lymphocytic leukemia,^28-31^ mantle cell lymphoma,^32^^,^^33^ and a subset of B-ALL.^34^^,^^35^ It is, therefore, a promising target for novel immunotherapy approaches, especially as it is expressed on cancer-initiating cells, a subpopulation of cancer cells that are resistant to standard cancer therapies but capable of self-renewal and tumor recurrence.^36^^,^^37^ Furthermore, high ROR1 levels on tumor cells correlate with metastases and poor outcomes.^18-21^^,^^38^ ROR1 is absent on all critical organs but is expressed at low level on adipocytes and parts of the gut, pancreas, and parathyroid glands.^14^ Importantly, CAR T cells and a monoclonal antibody directed against ROR1 have not demonstrated any toxicity in animal models or humans.^39^^,^^40^ However, BiTEs targeting ROR1 remain untested to date.

In this study, we describe the development and characterization of a BiTE that targets ROR1. Our ROR1 BiTE mediated antigen-specific cytotoxicity across a range of solid tumor cells including pancreatic cancer cell lines with concurrent cytokine production *in vitro*. In murine models, ROR1 BiTE prevented the engraftment of pancreatic and ovarian cancer cells in xenograft models and reduced the size of established subcutaneous pancreatic tumors. Humanization of the binding arms, to minimize immunogenicity, did not abrogate its effector function. Therefore, our ROR1-BiTE provides a novel platform for T-cell-mediated targeting of a range of solid tumors.

## Materials and methods

### Single chain variable fragment generation

Rats were immunized against the extracellular portion of ROR1 by Aldevron GmBH. Oligoclonal clones from the subsequent hybridomas were single cell sorted and immunoglobulin heavy and light chain sequences were isolated by 5′ reverse amplification of cDNA ends (5′ RACE) using the standard laboratory protocols. Productive sequences, as identified by the International Immunogenetics Information System V-Quest tool,^41^ were cloned in frame with heavy and light chain constant regions, and antibodies were generated by transient co-transfection. Specific binding for ROR1 was demonstrated before the conversion of the variable domains to scFvs in a heavy chain-linker-light chain format.

### Cell lines and reagents

PANC-1, SKOV-3, and HEK293T cells were obtained from American Type Culture Collection (ATCC; LGC Standards). MCF7 cells were obtained from Deutsche Sammlung von Mikroorganismen und Zellkulturen GmbH. SUIT-2, CFPAC1, HPAF-II, MiaPaCa2, and PSN-1 cell lines were kindly provided by Professor Aldo Scarpa (Department of Pathology and Diagnostics, University and Hospital Trust of Verona, Verona, Italy). Other cell lines were from master cell banks within our institute. HEK293T cells were maintained in Iscove's Modified Dulbecco's Medium (ThermoScientific) supplemented with 10% fetal bovine serum (FBS) (ThermoScientific). All the other cell lines were maintained in RPMI-1640 medium (ThermoScientific) supplemented with 10% FBS, GlutaMAX, and 25 mM HEPES. Cells were cultured at 37°C with 5% CO_2_. Cell lines were screened for mycoplasma to ensure negativity before functional assessment. ROR1 expression was assessed using a commercial anti-ROR1 antibody (Clone 2A2, Biolegend) by flow cytometry.

### BiTE generation

The ROR1 and control CD19 fmc63 scFvs were coupled to the anti-human CD3 scFv (Clone OKT3) through a short amino acid linker using gBlocks (Integrated DNA Technologies) and overlapping extension PCR using Phusion DNA polymerase (New England Biolabs). The BiTE open reading frame (ORF) was cloned into the SFG retroviral vector upstream of a GFP ORF, by NcoI/MluI restriction digestion. The two ORFs are separated by an IRES region to obtain the SFG.ROR1-BiTE.IRES.GFP or SFG.CD19-BiTE.IRES.GFP. To allow for the purification and detection of the BiTEs, we included an N-terminal hexa-Histidine Tag.

### Generation of HEK293T stable BiTE producer cells

Retroviral supernatant was produced in HEK293T cells using the RD114 retrovirus envelope (RDF), PeqPam3 gag-pol, and SFG.ROR1-BiTE.IRES.GFP or SFG.CD19-BiTE.IRES.GFP transfer vectors following the standard laboratory protocols. Supernatants containing retroviral vectors were harvested 48 and 72 h after transfection, immediately frozen on dry ice and stored at −80°C until further use. HEK-293T cells (1.8 × 10^6^) were plated in 10 cm dishes in fresh media, and transduced with 2 mL of supernatant containing retrovirus at 24 and 48 h post-seeding. Transduced cells were incubated for 72 h in a humidified incubator at 37°C with 5% CO_2_ and sorted based on GFP expression and tested for BiTE production.

### BiTE production, purification, and binding

HEK293T media containing BiTEs was collected and purified by fast protein liquid chromatography (FPLC) using HiTrap Talon binding columns with an AKTA Explorer (GE Healthcare Life Sciences). The quality of the BiTE purification was assessed by Coomassie staining after SDS-PAGE and quantified using Pierce bovine serum albumin (BSA) standard dilutions (ThermoScientific). ImageJ software was used for data analysis (the U. S. National Institutes of Health). BiTEs were validated by western blot using an horseradish peroxidase (HRP) conjugated anti-His antibody (Biolegend). The specific binding of ROR1 BiTE or CD19 BiTE to target cells was assessed by flow cytometry using an anti-His Tag antibody (Abcam). Size exclusion chromatography was undertaken with a Shimadzu Nexera XR High-performance liquid chromatography (HPLC) machine and Waters size exclusion chromatography column (Bio-Analysis Center London).

### T cell purification

Peripheral blood mononuclear cells from healthy donors were obtained after the centrifugation of fresh isolated volunteer blood or buffy coats (NHS blood and transplant) on a density gradient using Ficoll-Paque Plus (GE Healthcare Life Sciences). In keeping with previous reports, freshly isolated T cells were expanded for animal work only: T cells were plated at 1 × 10^6^ cells per well in 24-well plates and expanded using CD3/CD28 beads (ThermoScientific) or 200 IU/mL of IL-2 (Miltenyi), and kept in culture for between 24 h and 1 week before injecting into mice. Freshly isolated unstimulated T cells were used for all *in vitro* experiments.

### Flow cytometry

Data were captured on an LSR Fortessa II flow cytometer (Becton Dickinson) and analyzed using FlowJo software (Flowjo LLC). Fluorescence activated cell sorting was undertaken on a FACSAria Cell Sorter (Becton Dickinson).

### Co-cultures assay

Co-culture assays were performed in 96-well plates, containing 1 × 10^4^ target cells, 1 × 10^4^ T cells, and purified BiTE at a concentration of 0.1 ng/mL–1 µg/mL. Twenty-four hours after the addition of ROR1 BiTE or CD19 BiTE, supernatant was collected for cytokine evaluation, which was performed by ELISA following the manufacturer's instructions (Biolegend). To assess cytotoxicity, we used the CellTiter 96 AQueous One Solution Cell Proliferation Assay (MTS) following the manufacturer's protocol (Promega).

### Immunohistochemistry

The heavy and light chains of our ROR1 scFv were cloned in frame with the murine IgG1 constant and kappa constant regions, respectively, and antibody was obtained from Absolute Antibody Ltd. Normal pancreas and pancreatic tissue microarrays were obtained from US-Biomax. Slides were prepared using the standard laboratory protocols. Briefly, antigen retrieval was undertaken by immersing slides in 0.01 M sodium citrate buffer, pH 6.0 at 95°C for 15 min before cooling and rinsing once with PBS, and then blocked and stained with ROR1 antibody (1:250) in PBS/Tween20, 0.05% BSA, 1% NaN_3_ 4 mM for 60 min at room temperature. Slides were incubated with the HRP-conjugated secondary, Histofine Simple Stain MAX PO (Nichirei), and developed using Stable DAB Plus (Diagnostic Biosystems).

### Humanization

The variable domain sequences of rat-derived ROR1 and mouse-derived CD3 scFvs were searched against a human IgG germline database. A human framework sequence with high homology to rat or mouse antibody was chosen as human acceptors for both light and heavy chains and humanized scFv and antibodies were assessed for a specific binding against ROR1 positive and negative cell lines.

### Statistics

Statistical analysis was undertaken using appropriate statistical tests in GraphPad Prism Version 6 for Windows. Statistical significance was taken when *p* < 0.05 and error bars represent standard deviation. At least two independent experiments with different donor T cells were undertaken for all *in vitro* experiments.

### Animal studies

All animal works were performed under the authority of the United Kingdom Home Office Project and Personal License regulations and were compliant with University College London guidelines. Six- to eight-week-old female Hsd:Athymic Nude-Foxn1^nu^ mice (Charles Rivers Laboratories) received 2 × 10^6^ PANC-1.Luc or 5 × 10^6^ SKOV3.Luc cells by intraperitoneal injection. PANC-1.Luc and SKOV3.Luc luciferase expression was detected using D-Luciferin (Melford Laboratories), which was injected intraperitoneally (IP) at a dose of 200 µg/mouse, and imaged using the IVIS Imaging System 100 Series (Perkin Elmer) at multiple time points. Living Image 4.4 software (Perkin Elmer) was used to quantify bioluminescence imaging (BLI) signal. For xenograft studies, 5 × 10^6^ of PANC-1 cells were mixed in an equal volume of Matrigel (Corning) and were injected in the flank of 6–8-week-old Hsd:Athymic Nude-Foxn1^nu^. Once the xenograft were established (minimum size 100 mm^3^), mice received 5 × 10^6^ T cells by single tail vein injection, followed by a daily injection of PBS, ROR1, or CD19 BiTE suspended in 0.1% BSA in PBS (10 µg/kg/mouse). Tumor volume was calculated using the ellipsoidal formula (length × width^2^)/2.

## Results

### Design, expression, and purification of ROR1 BiTE

**Figure 1.**
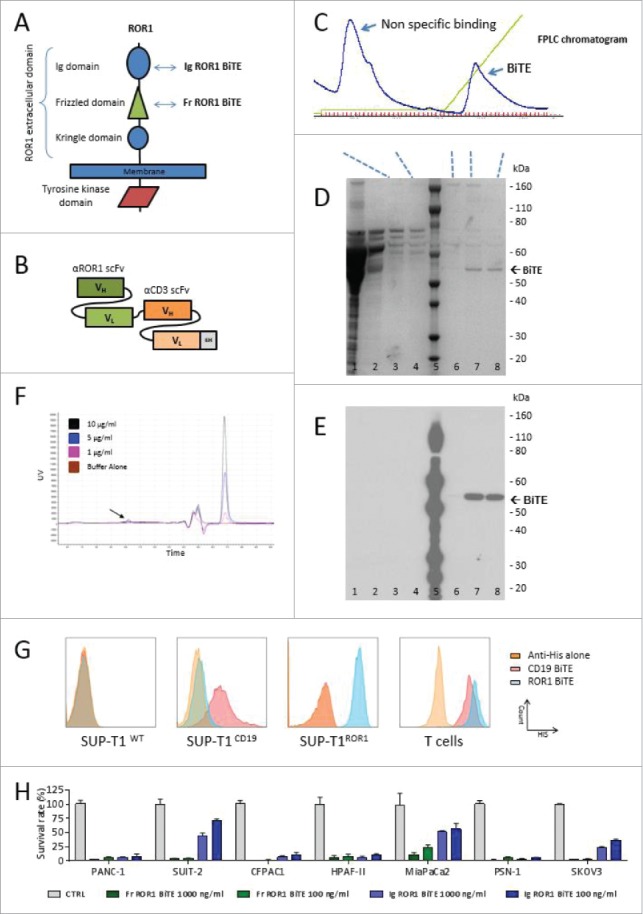
ROR1 BiTE design, characterization, and production. (A) Schematic representation of ROR1. The extracellular domain of human ROR1 is composed of three domains: Immunoglobulin like, Frizzled, and Kringle domains. A panel of antibodies were isolated that targeted either the membrane distal immunoglobulin or more membrane proximal frizzled domain. (B) Schematic of BiTE. Heavy chain variable regions (VH) and light chain variable regions (VL) are linked together to create single chain variable fragments (scFv), the first is specific for the ROR1, and the second for human CD3, separated by a short flexible linker. A C-terminal hexa-Histidine Tag allowed for detection and purification. (C) Purification of BiTEs was undertaken with a three-step imidazole elution protocol via FPLC. This yielded an initial large peak corresponding to non-specific binding to the HiTrap column followed by isolation of purified BiTE. (D) and (E) Coomassie staining of SDS PAGE showing the different protein presents in each peak and representative western blot analysis of eluted fractions using anti-His Tag antibody (Lanes: 1 crude supernatant, 2 Flow-through, 3 and 4 Non-specific binding peak, 5 protein ladder, 6 Pre-BiTE-peak fraction, 7 and 8 BiTE peak fractions). (F) Size exclusion chromatography HPLC of ROR1 BiTE at three different concentrations (1, 5 and 10 µg/mL) showing <5% aggregation (black arrow) compared with buffer alone. The double peak before main fraction (black star) represents the buffer peak (see maginified version in Fig. S1). (G) Representative Binding Assessment of BiTEs. Binding was assessed against a panel of SUP-T1 modified cells lines or T cells and detected with an anti-His antibody. The CD3, ROR1, and CD19 negative SUP-T1^WT^ showed no binding with ROR1 or CD19 BiTEs. SUP-T1 cells engineered to expressed either ROR1 (SUP-T1^ROR1^) or CD19 (SUP-T1^CD19^) showed binding with ROR1 and CD19 BiTE, respectively. To assess binding of the CD3 scFv, we used purified T cells and incubated them in the presence of BiTEs. (H) Following purification, quantification, and normalization, cytotoxicity with immunoglobulin domain binding ROR1 BiTEs was compared against Fr-ROR1xCD3 at two different concentrations (100 ng/ml or 1000 ng/ml) in a killing assay where target cells and T cells were cultured in a 1:1 effector:target ratio. Cell viability was assessed at 24 h.

We isolated a panel of anti-ROR1 antibodies that bound to either the membrane distal immunoglobulin like (Ig) or the more proximal frizzled (Fr) domain of ROR1 from a rat hybridoma library (Fig. 1A). When converted into an scFv format, they retained ROR1-specific binding and were coupled to a murine CD3 scFv in a tandem structure separated by a short five-amino acid (Gly_4_Ser) linker to create an ROR1 BiTE molecule (Fig. 1B). BiTEs were stably expressed in HEK-293T cells following gene transfer with retroviral vectors. Supernatant harvested from these producer cells was purified using metal-affinity chromatography with a distinct peak observed at approximately 40 mM imidazole. SDS/PAGE electrophoresis showed a dominant band at 52 kDa, corresponding to the expected molecular weight of the BiTEs, with the verification of the BiTE confirmed by Western blot analysis, respectively (Fig. 1C–E). BiTEs can aggregate with storage affecting their binding characteristics and effector function. To assess this, we undertook independent size exclusion chromatography HPLC, demonstrating <5% aggregation of the purified BiTE at multiple dilutions (Fig. 1F, Fig. S1). Flow cytometry staining of the SUP-T1 cell line engineered to express either human CD19 or ROR1 confirmed specificity of the CD19 and ROR1 BiTE, while human T cells showed specific binding to CD3 with both CD19 and ROR1 BiTEs (Fig. 1G). Head-to-head comparison in a panel of ROR1 positive cancer cell lines demonstrated that the ROR1 BiTE containing an scFv directed against the Fr domain yielded consistently superior and reproducible cytotoxicity compared with BiTEs targeting the Ig domain (Fig. 1H). Therefore, this Frizzled domain scFv was selected for further assessment.

### ROR1 expression in pancreatic cancer and normal human tissues from critical organs

**Figure 2.**
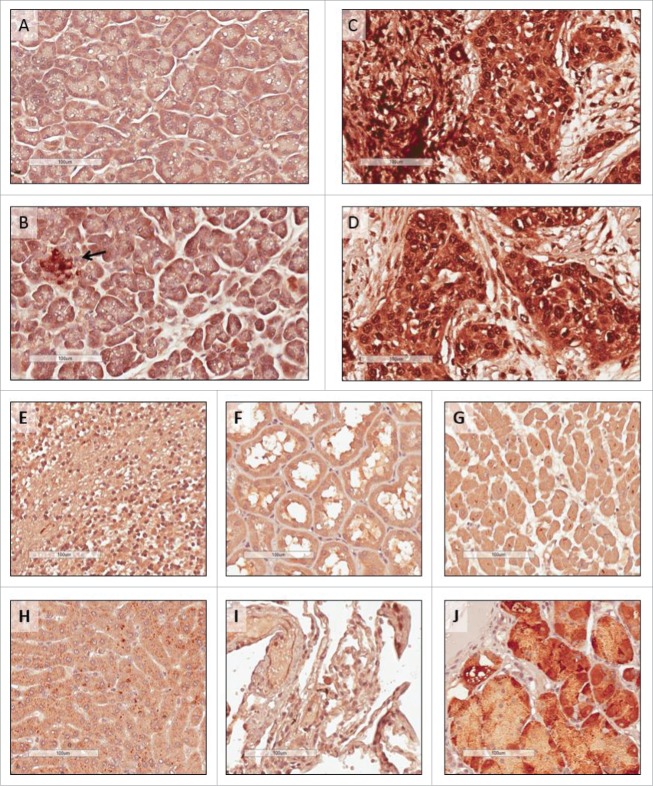
Immunohistochemistry staining of human tissues. Tissue microarrays were stained with anti-human ROR1 antibody with the same specificity and antigen binding arms as the BiTE. (A) and (B) Normal pancreas, with islet cells highlighted (arrow); (C) and (D) pancreatic cancer from two independent patients; (E) normal brain; (F) normal kidney; (G) normal heart; (H) normal liver; (I) normal lung, and (J) normal stomach. All scale bars: 100 μm.

We converted our ROR1 scFv into a full antibody to allow the assessment of ROR1 expression in human tissue microarrays and resected tumors by immunohistochemistry using the identical binding domain as used in the ROR1 BiTE. High ROR1 expression was detected in pancreatic cancer specimens with positive staining localized to the cytoplasm as well as the nucleus of cancer cells (Fig. 2C and D). In contrast, only the islet cells stained for ROR1 in samples of normal pancreatic tissues (Fig. 2A and B). ROR1 expression was also observed in regions of the stomach (Fig. 2J), consistent with previous reports.^14^ Importantly, ROR1 was not detected in normal tissue samples from the heart, liver, brain, kidney, or lungs (Fig. 2E–I).

### ROR-BiTE directs T cells to kill ROR1-expressing pancreatic cancer cells in vitro

**Figure 3.**
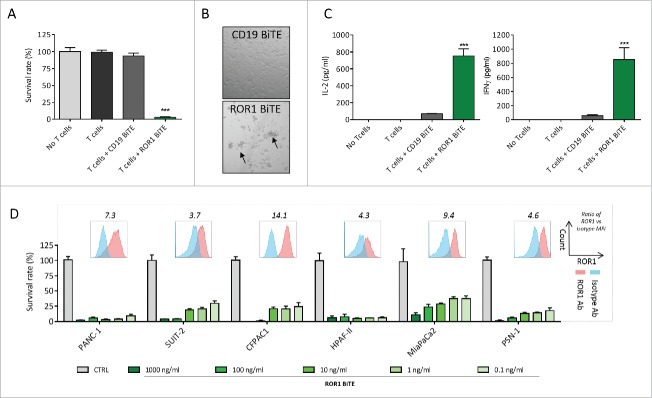
ROR1 BiTE mediates specific cytotoxicity against pancreatic ductal adenocarcinoma (PDAC) cell lines *in vitro*. (A) Cell viability assay demonstrates marked cytotoxicity against PANC-1 cell lines with ROR1 BiTE and unstimulated T cells but not with T cells alone or T cells with control CD19 BiTE (1:1 effector:target ratio; 1 μg/mL BiTE). (B) T-cell clustering was seen when T cells were placed in the presence of target PANC-1 cells and ROR1 but not CD19 BiTE (CTRL). (C) IL-2 and IFNγ secretion by T cells in response to PANC-1 cells in the presence of ROR1 or CD19 BiTE. (D) Dose-dependent cytotoxicity with ROR1 BiTE in co-culture assays using a panel of PDAC cell lines, which retains its cytotoxic activity at concentrations as low as 0.1 ng/mL. Flow cytometry analysis of ROR1 staining on cell lines compared with isotype control shown for each cell line, values represent fold increase of MFI value compared with isotype control.

We next sought to assess the ability of our ROR1 BiTE to elicit antigen-specific cytotoxic responses *in vitro*. In a standard cell viability assay, unstimulated T cells were co-cultured with the ROR1 positive PANC1 pancreatic ductal adenocarcinoma cell line at a 1:1 effector:target ratio (E:T) in the presence of either ROR1 BiTE or control CD19 BiTE. The ROR1 BiTE demonstrated significant cytotoxicity with the killing of 97.3% of cells (*p* < 0.001) (Fig. 3A) associated with T cell proliferation and clustering (Fig. 3B) and a 15-fold and 11-fold increase in interferon-γ (IFNγ) and interleukin-2 (IL-2) secretion, respectively (Fig. 3C). Target cell lysis or cytokine release was not observed upon the incubation of pancreatic cancer cells with ROR1 BiTE in the absence of T cells (Fig. 3A, C, and D, designated “No T cells”), demonstrating the need for dual specificity for killing of tumor targets. Additionally, we did not observe any cytotoxicity in co-cultures of PANC-1 cells with T cells without ROR1 BiTE or in the presence of control CD19 BiTE. The latter is consistent with the lack of CD19 expression on pancreatic cancer cells. Dose-dependent killing was observed across a panel of ROR1 positive pancreatic cell lines comprising SUIT-2, CFPAC1, HPAF-II, MiaPaCa2, and PSN-1 with our ROR1 BiTE (mean cytotoxicity at 1 μg/mL, 96%). Significant T-cell-mediated killing was observed even at concentrations of 0.1 ng/mL (Fig. 3D). However, T-cell-mediated killing did not correlate with ROR1 expression on target cells.

### Antitumor response of ROR1 BiTE in two distinct murine xenograft models

**Figure 4.**
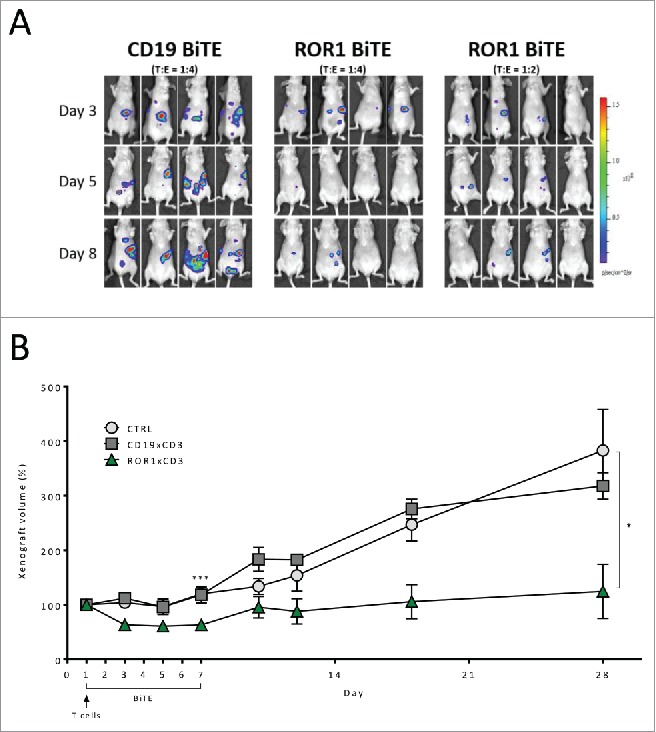
*In vivo* assessment of ROR1 BiTE using luciferase positive PANC1 cell line. (A) Intraperitoneal Engraftment: 2 × 10^6^ PANC-1.Luc cells/mouse were administered followed by a single dose of purified human T cells injection intraperitoneally (8 × 10^6^ CTRL BiTE group; 8 × 10^6^ and 4 × 10^6^ ROR1xCD3 BiTE groups 1 and 2, respectively). BiTEs were injected daily at 10 μg/kg/mouse for 5 d. PANC-1.Luc engraftment was assessed by *in vivo* bioluminescent imaging (BLI). (B) Established xenograft model: PANC-1 cell lines (5 × 10^6^) were injected in the right flank of immunocompromised athymic nude mice and xenografts were established to a minimum size of 100 mm^3^. Mice then received a single intravenous injection of purified T cells (5 × 10^6^) and were treated with ROR1 BiTE, CD19 BiTE or PBS at 10 μg/kg/d iv daily for 7 d and the size of the tumor measured by caliper. Follow-up of these animals at day 28 showed that ROR1 BiTE treated mice had lower tumor volumes compared with the control cohorts despite no additional ROR1 BiTE administration.

Currently, available animal models of pancreatic cancer are limited in their reproduction of the complex tumor environment. However, to provide *in vivo* proof of concept, we assessed our ROR1 BiTE in two murine xenograft models. First, we injected 2 × 10^6^ PANC-1, firefly luciferase-positive cells (PANC-1.Luc) into the peritoneal-cavity of athymic nude mice followed by a single bolus administration of either 4 × 10^6^ (E:T ratio of 2:1) or 8 × 10^6^ (E:T ratio of 4:1) human T cells. Mice received a daily intraperitoneal injection of ROR1 or control CD19 BiTEs (10 µg/kg/mouse) for 5 d. Analysis on day 8 revealed PANC-1.Luc engraftment, as assessed by non-invasive bioluminescence imaging (BLI), was reduced in a T-cell-dose-dependent manner by 11- or 15-fold in the 2:1 and 4:1 cohorts of mice, respectively, compared with animals that received CD19 BiTE (Fig. 4A, Fig. S2). We next undertook a subcutaneous model in which 5 × 10^6^ PANC-1 cells were injected into the right flank of athymic nude mice and were allowed to establish to a minimum size of 100 mm^3^. Animals subsequently received a single tail vein administration of 5 × 10^6^ purified T cells, followed by the intraperitoneal administration of ROR1 BiTE at a dose of 10 µg/kg/mouse daily for 7 d without exogenous cytokine support. Control cohorts received either CD19 BiTE at an identical dosing regimen or excipient (PBS). Treatment with ROR1 BiTE reduced the growth of xenografts by greater than 50% during the treatment period as assessed after 7 d of ROR1 BiTE therapy when compared with control animals (mean size 62.2 mm^3^ vs. 119.7 mm^3^, *p* = 0.003). Longer follow-up showed that despite only 7 d of ROR1-BiTE therapy and a single infusion of T cells, the ROR1 BiTE-treated cohort maintained lower tumor volumes compared with the control mice (Day 28:mean size 171.2 mm^3^ vs. 361.2 mm^3^, *p* = 0.037), suggesting transient treatment with ROR1 BiTE can lead to a durable antitumor responses (Fig. 4B).

### ROR1 BiTE humanization

**Figure 5.**
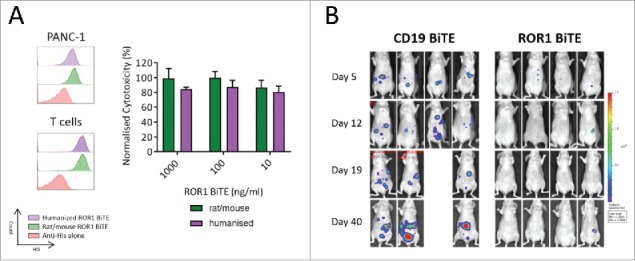
Fully humanized ROR1 BiTE *in vitro* and *in vivo* efficacy. (A) Rat/Mouse non-humanized ROR1 BiTE and fully humanized ROR1 BiTE showed equivalent affinity for ROR1 and CD3 against PANC1 positive target cells. Humanized ROR1 BiTE led to equivalent cytotoxicity against PANC1 target cells and T cells compared with parental non-humanized BiTE. (B) Mice received 2 × 10^6^ PANC-1.Luc cells/mouse intraperitoneally followed by three doses of purified 4 × 10^6^ human T cells. Humanized BiTE was injected once per week at 10 µg/kg/mouse and PANC-1.Luc engraftment was assessed by *in vivo* bioluminescent imaging (BLI).

Our BiTE is composed of a rat anti-ROR1 and murine anti-CD3 scFvs, leading to the potential for anti-BiTE-mediated immune responses, potentially limiting its clinical efficacy. This is especially true as normal CD19 positive B cells are ROR1 negative and would be spared, thus allowing production of neutralising antibodies. In view of this, we undertook a humanization program and grafted the rat and murine complementarity-determining regions of the parental scFvs onto highly homologous acceptor human framework regions. The antigen-binding characteristics, production, purification, and killing of ROR1 positive targets of the humanized BiTEs were comparable to the original rat-mouse hybrid ROR1-CD3 BiTE (Fig. 5A) with no evidence of loss of specificity when assessed against ROR1 negative cell lines (data not shown)**.** To confirm *in vivo* efficacy of our humanized ROR1 BITE, mice received 2 × 10^6^ PANC1.Luc cells intraperitoneally and were then treated with once weekly T cells (8 × 10^6^ human T cells; E:T ratio of 4:1) and fully humanized ROR1 BiTE. As with the non-humanized BiTE, we saw marked tumor reduction by day 8. Mice were subsequently treated with weekly BiTE injections, with extended follow up to 40 d confirming long-term efficacy (Fig. 5B, Fig. S2).

### In vitro and in vivo antitumor efficacy of ROR1 BiTE across a range of solid tumor lines

**Figure 6.**
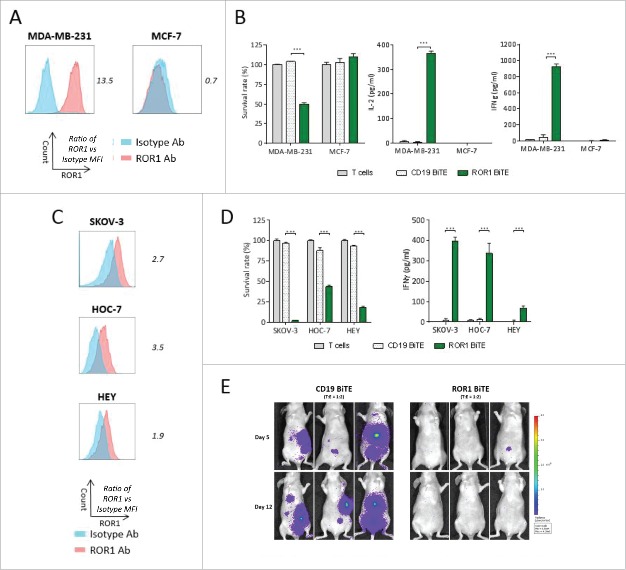
ROR1 BiTE provides *in vitro* and *in vivo* specific cytotoxicity against breast and ovarian cancer cell lines. (A) Flow cytometry staining of ROR1 on MDA-MB-231 cells lines and MCF7 breast cancer cell lines compared with isotype control, values represent fold increase in MFI value. (B) Cytotoxicity and cytokine secretion against ROR1 positive MDA-MB-231 but not ROR1 negative MCF7 cell lines as assessed by a cell viability assay using 1 μg/mL ROR1 BiTE at 24 h compared with CD19 BiTE (1:1 Effector: Target ratio). (C) Flow cytometry staining of ROR1 on SKOV3, HOC-7, and HEY ovarian cancer cells lines compared with isotype control, values represent fold increase in MFI value. (D) Cytotoxicity and IFNγ secretion of ovarian cancer cell lines as assessed by a cell viability MTS assay using 1 μg/mL ROR1 BiTE at 24 h compared with CD19 BiTE. (E) 5 × 10^6^ SKOV-3.Luc cells/mouse were administered followed by a single dose of purified 10 × 10^6^ human T cells injection intraperitoneally. BiTEs were injected daily at 10 μg/kg/mouse for 5 d. SKOV3.Luc engraftment was assessed by BLI.

We next sought to test the ability of ROR1 BiTE to elicit antigen-specific cytotoxic responses *in vitro* against other solid tumor cells lines. T cells and ROR1 BiTE were co-cultured with either ROR1 positive MDA-MB-231 or ROR1 negative MCF-7 breast cancer cell lines. Cytotoxicity and cytokine secretion were only observed with the former, confirming the presence of ROR1 was essential for T cell activation (Fig. 6A and B). Importantly, there was no evidence of T cell activation when ROR1 BiTE was cultured with T cells in the absence of ROR1 positive target cells. We also assessed cytotoxicity against SKOV-3, HOC-7, and HEY ovarian cancer cell lines that express marginally different levels of ROR1 with killing and cytokine release observed against all cell lines (Fig. 6C and D) but as before, did not observe a correlation between ROR1 expression and cytotoxicity. *In vivo* efficacy was assessed in athymic nude mice injected intraperitoneally with SKOV-3 cells expressing firefly luciferase (SKOV-3.Luc) together with a single administration of T cells in an E:T ratio of 2:1 ratio. Mice were treated for 5 d with ROR1 BiTE, or control CD19 BiTE. The ROR1 BiTE prevented engraftment of SKOV-3.Luc cells compared with the CD19 BiTE treated animals that had significant tumor burden at day 12 (Fig. 6E, Fig. S2). Finally, we tested our ROR1 BiTE *in vitro* against a panel of ROR1 positive cell lines representing melanoma (T618A), glioblastoma (U-251, A 172), prostate (DU145, PC-3), and hepatic cancer (SK-Hep-1, HUH7) and confirmed significant cytotoxicity against all of these ROR1 positive cell lines, demonstrating wider applicability in targeting a range of tumor subtypes (Fig. S3).

## Discussion

From a panel of rat anti-human ROR1 antibodies, we selected an scFv that targeted the cysteine-rich frizzled domain of ROR1 as it consistently yielded superior and reproducible cytotoxicity compared with scFv that targeted the ROR1-immunoglobulin-like domain. In keeping with this, a bispecific antibody targeting the membrane proximal domain of FcHR5 showed superior cytotoxicity compared with one targeting a membrane distal domain, and this was shown to be due to more efficient cytotoxic synapse formation with the former.^42^ Our resulting ROR1 BiTE facilitated efficient T-cell-mediated killing of pancreatic and ovarian cancer *in vitro* and *in vivo* as well as a range of solid tumor cell lines of different histological origins. Variation in ROR1 expression between tumor types, as well as within cells lines derived from tumors of the same histological origin, was observed. However, our ROR1 BiTE was able to mediate efficient and equivalent killing of tumor cells with low and high levels of ROR1 with relatively low effector to target ratio. Optimal T cell activation required dual specificity as our ROR1 BiTE could not elicit functional T cell activation or cytokine release in the absence of engagement with ROR1, as illustrated with the ROR1 negative MCF7 cell line. Interestingly, a relatively low concentration of ROR1 BiTE was required (nanogram quantities) when compared with doses (microgram quantities) of conventional antibodies used in clinic, to mediate killing of tumor targets. This indicates that the avidity gained by bispecific binding, in combination with the large signal amplification by the T cell receptor, enhanced the potency of this bi-specific molecule.^43^ Moreover, to minimize against potential immunogenicity, we undertook a humanization program of both the ROR1 and CD3 scFvs and developed a fully humanized BiTE that facilitates equivalent cytotoxicity against target cell lines *in vitro* and retains its effector function *in vivo*.

Our study confirms previous findings that ROR1 is expressed on some normal tissues,^14^ which raises the possibility of on-target off-tumor toxicities. However, Cirmtuzumab, a humanized high affinity anti-ROR1 monoclonal antibody directed toward the immunoglobulin-like domain of ROR1, has been safely administered to 12 patients with CLL with no significant toxicity reported (NCT02222688).^39^^,^^40^ Plans are in place to evaluate the same antibody in patients with breast cancer (NCT02776917). In addition, the administration of relatively high dose of functional ROR1-CAR T cells (5 × 10^8^ T cells/kg) in non-human primates did not result in any toxicity, despite similar levels and patterns of ROR1 expression in non-human primates compared with humans.^39^ Two ROR1 CAR T-cell Phase I/II studies have been registered for patients with hematological and solid malignancies (NCT02194374, NCT02706392).

These data are reassuring but our plan is to undertake a cautious evaluation of our own humanized ROR1-BiTE entailing a detailed independent immunohistochemistry study to exclude the potential of unidentified cross reactivity of our scFvs with normal human tissues. This would be followed by the systematic evaluation of high ROR1 BITE doses in non-human primate toxicology study before progressing to humans.

Given their promise, BiTEs against a range of tumor associated antigens have been generated^9^ including CD20,^44^ B-Cell maturation antigen (BCMA),^45^ epithelial cell adhesion molecule,^46^ and carcinoembryonic antigen (CEA),^47^ but the majority of clinical evaluation has so far been focused on patients with B cell malignancies with Blinatumomab. A number of trials with BiTEs against other targets are ongoing including BCMA (NCT02514239), CEA (NCT02291614), and PSMA (NCT01723475). Blinatumomab has resulted in high response rates in groups of patients who otherwise carry a poor prognosis including those with relapsed/refractory B-ALL or high-grade B cell non-Hodgkin's lymphoma.^11^^,^^12^ CD19 targeting CAR T-cells have demonstrated similar high levels of efficacy, thus providing clinicians with a choice of immunotherapies for patients with B-cell malignancies. Direct comparison of the efficacy of CD19 BiTE when compared with CD19 CAR T-cells is not possible in the absence of randomized control trials as they are in different stages of development. In addition, published reports often have small patient numbers with variable levels of pre-treatment disease burden. Cytokine release syndrome (CRS) and neurologic toxicity, the two main worrying complications, have been observed with both Blinatumomab and CD19 CAR T-cells therapies. The incidence of CRS with BiTEs may be lower, based on the published reports.^48^ Additionally, the ability to increase the dose of BiTEs in a stepwise manner and switch off the infusion, should toxicity occur, provides BiTEs with a theoretical advantage over CAR T-cells, which can expand *in vivo* in an unpredictable manner.

A major disadvantage of CAR T-cell therapy when compared with an off-the-shelf treatment like BiTEs is that it requires a complex, multi-step, labor intensive *ex-vivo* T-cell manufacturing process that is patient specific. The final composition CAR T-cells are often heterogeneous and vary in the number of gene modified T cells obtained, as well as specific cell composition and fitness.^49^ Furthermore, access to CAR T-cells is limited to a relatively small number of academic centers, with overall manufacturing capacity at present being insufficient to meet demand. A key advantage of CAR T-cells is their persistence *in vivo*, potentially resulting in durable antitumor response, while the risk of relapse remains high upon discontinuation of BiTE therapy, especially in aggressive malignancies. However, the potential of targeting ROR1 expressed by cancer-initiating cells such as those derived from ovarian cancer and glioblastoma^36^^,^^37^ may mitigate the risk of relapse and raise the potential of a cure.

In summary, we provide the first systemic evaluation and proof of concept of an ROR1 BiTE and show T-cell-mediated efficacy against a panel of solid tumors, raising the prospects of targeting a range of tumor types. Immunotherapy for solid tumors faces several challenges; combinational therapy may be required to facilitate efficient T cell infiltration, effector function and reversal of the immunosuppressive microenvironment. However, our humanized ROR1 BiTE represents a rational therapy for cancer patients with high-unmet need such as those with aggressive tumors like pancreatic cancer and warrants further assessment in clinical trials.

## Supplementary Material

Supplementary_materials.zip
