# Supplementary material for: An ROR1 bi-specific T-cell engager provides effective targeting and cytotoxicity against a range of solid tumors
Source: Oncoimmunology. 2017 May 17;6(7):e1326437. doi: 10.1080/2162402X.2017.1326437 (PMC5543882; doi:10.1080/2162402X.2017.1326437)
Supplement: Supplementary_materials.zip [file koni-06-07-1326437-s001.zip › Supplementary figure 1-3.pptx]

## Slide 1
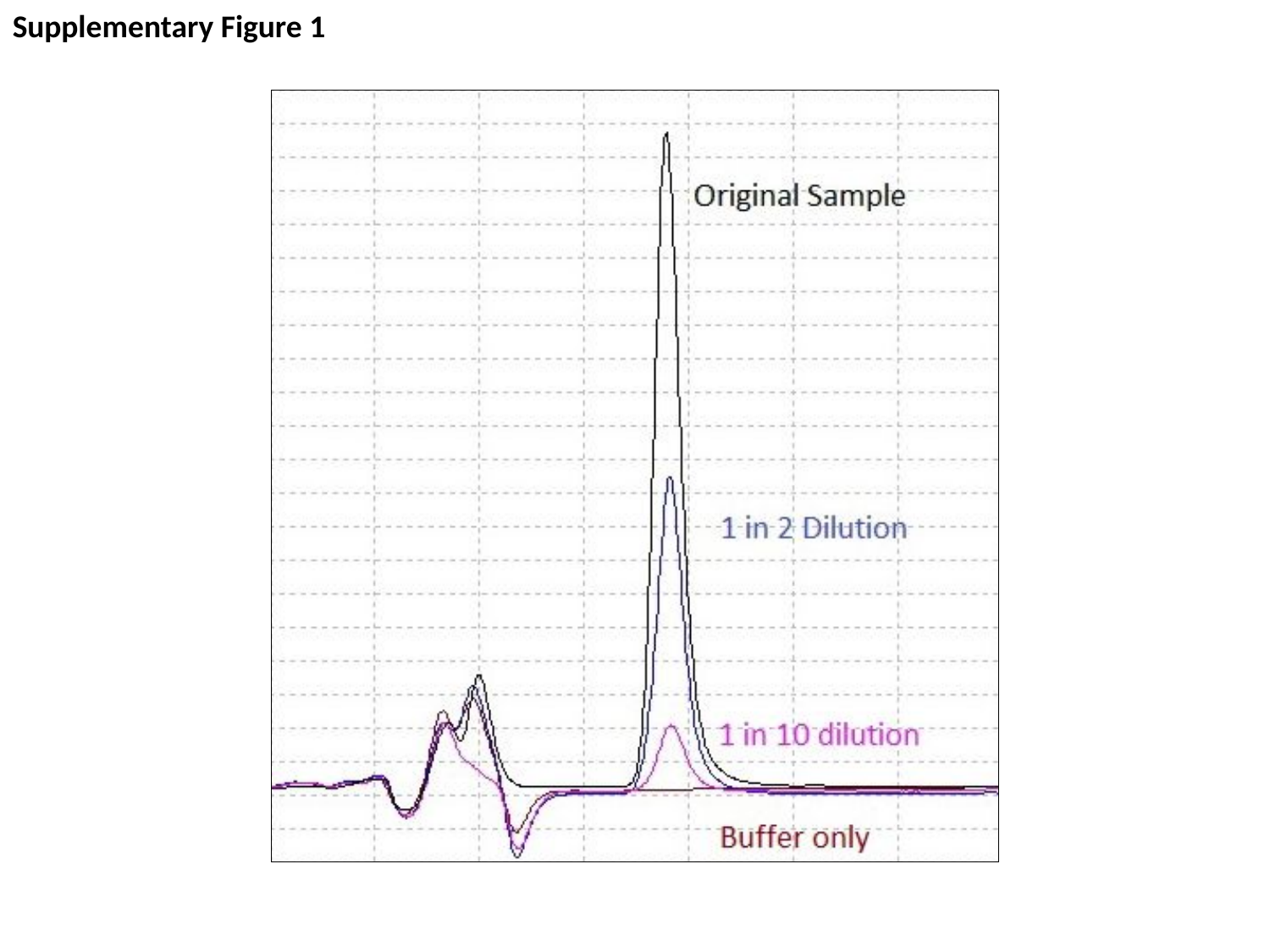

Supplementary Figure 1

## Slide 2
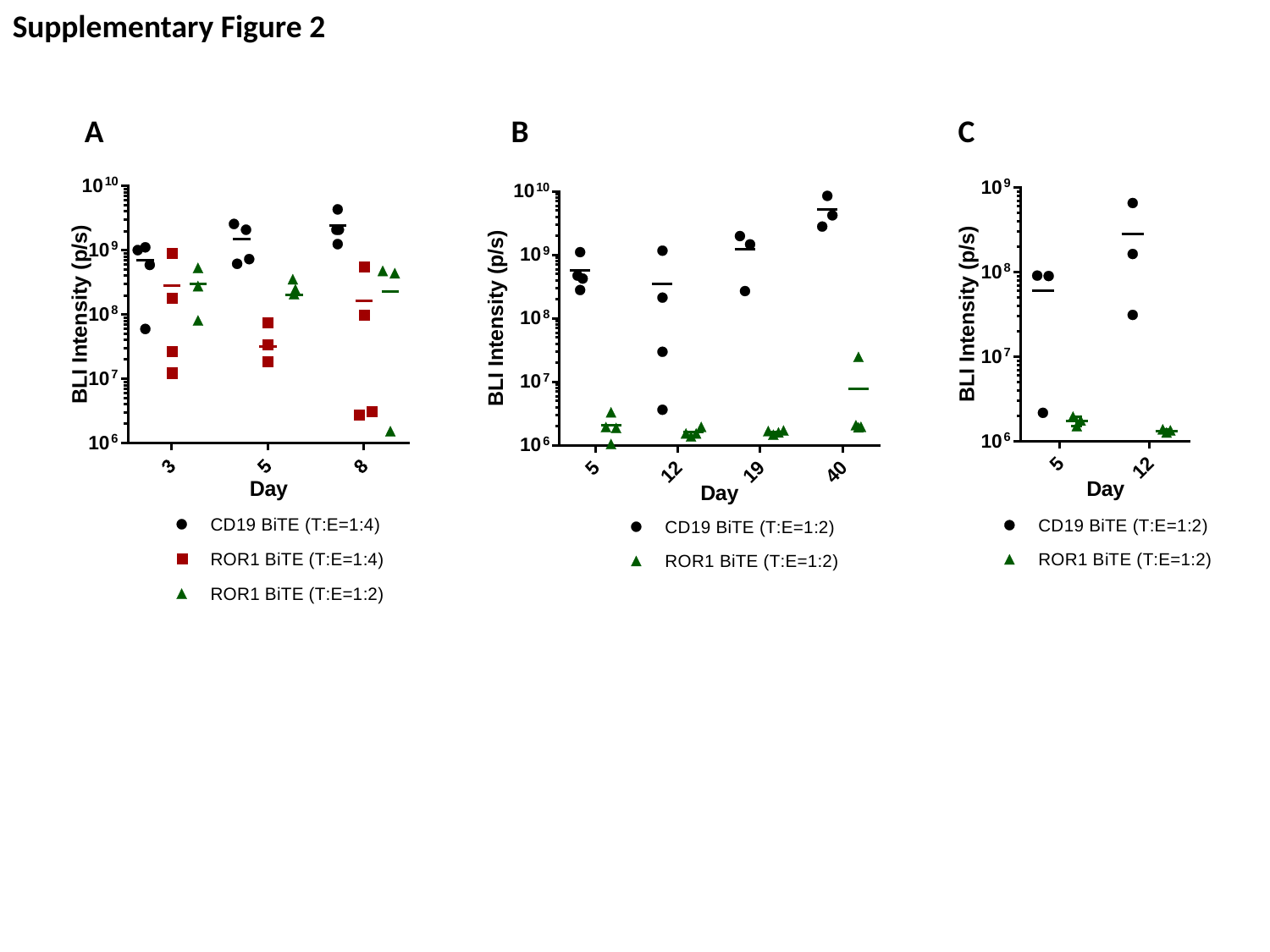

Supplementary Figure 2
A
B
C

## Slide 3
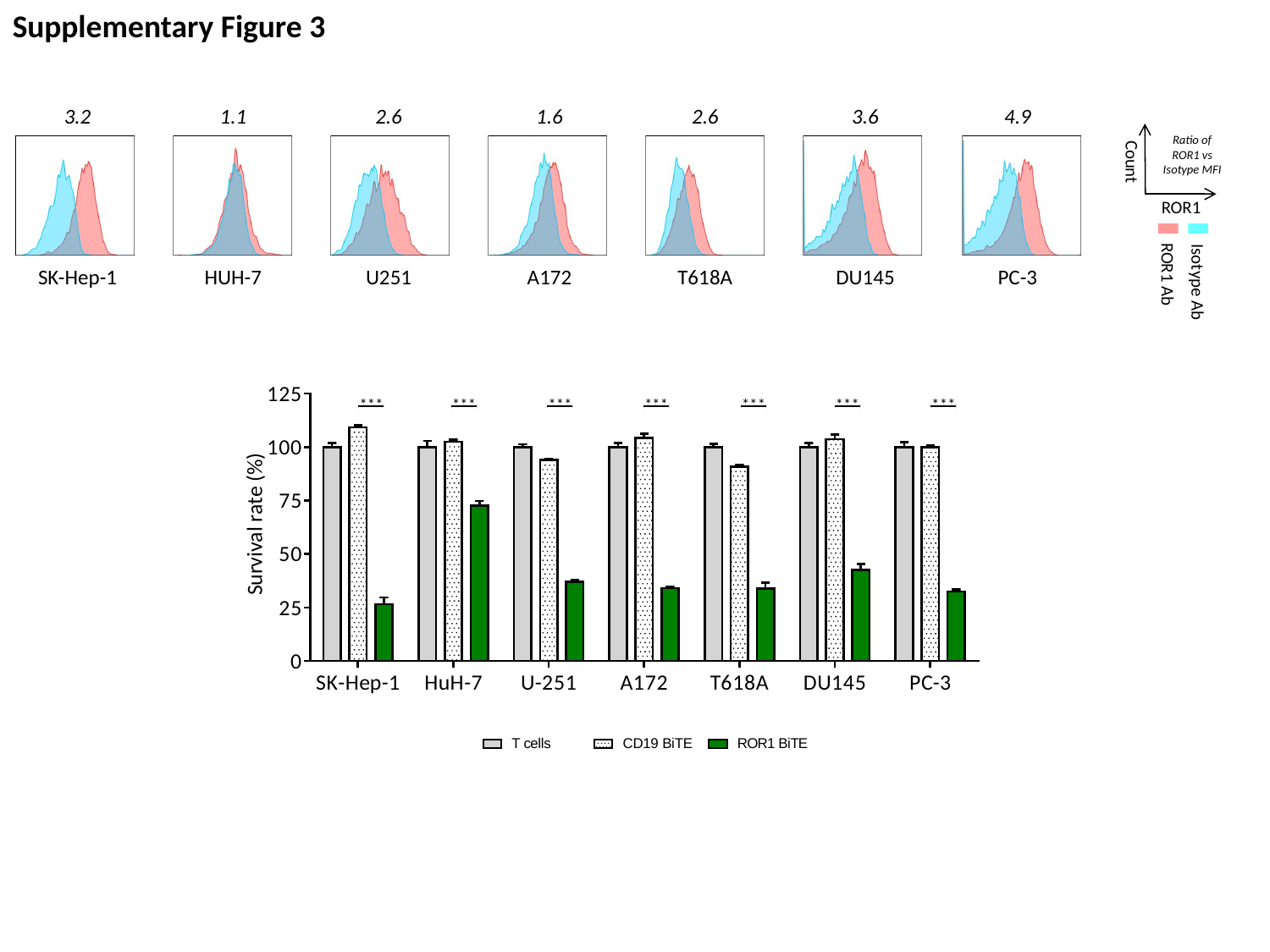

Supplementary Figure 3
3.2
1.1
2.6
1.6
2.6
3.6
4.9
Count
ROR1
Ratio of ROR1 vs Isotype MFI
Isotype Ab
ROR1 Ab
SK-Hep-1
HUH-7
U251
A172
T618A
DU145
PC-3
