# Supplementary material for: An ROR1 bi-specific T-cell engager provides effective targeting and cytotoxicity against a range of solid tumors
Source: Oncoimmunology. 2017 May 17;6(7):e1326437. doi: 10.1080/2162402X.2017.1326437 (PMC5543882; doi:10.1080/2162402X.2017.1326437)
Supplement: Supplementary_materials.zip [file koni-06-07-1326437-s001.zip › Supplementary legend.docx]

**Supplementary Figure 1: Size Exclusion Chromatography-HPLC**

Magnified view of Figure 1F to highlight the double peak. This corresponds to the buffer peak with no excess aggregation seen with the ROR1 BiTE.

**Supplementary Figure 2: Bioluminescence intensity in murine models**

Bioluminescence intensity of the whole mouse body was undertaken with region of interest analysis using Living Image 4.4 software.

A) PANC1.Luc intraperitonal model with BLI analysis of Figure 4A undertaken on Days 3, 5 and 8

B) PANC1.Luc intraperitonal model with BLI analysis of Figure 5B undertaken on Days 5, 12, 19 and 40.

C) SKOV3.Luc intraperitonal model with BLI analysis of Figure 6C undertaken on Days 5 and 12.

**Supplementary Figure 3: ROR1 BiTE enables targeting of a range of tumor subtypes.**

**A)** ROR1 expression was assessed on SK-Hep1, HUH7, U251, A172, T618A, DU145 and PC-3 cell lines representative of hepatic, glioblastoma, melanoma and prostate cancer; values indicate fold increase of MFI compared to isotype.

**B)** Cell viability assay demonstrated cytotoxicity against all positive cell lines compared with control CD19 BiTE (1:1 effector:target ratio; 1μg/ml BiTE).
