# Supplementary figures and images for: An ROR1 bi-specific T-cell engager provides effective targeting and cytotoxicity against a range of solid tumors
Source: Oncoimmunology. 2017 May 17;6(7):e1326437. doi: 10.1080/2162402X.2017.1326437 (PMC5543882; doi:10.1080/2162402X.2017.1326437)

Supplementary Figure 1

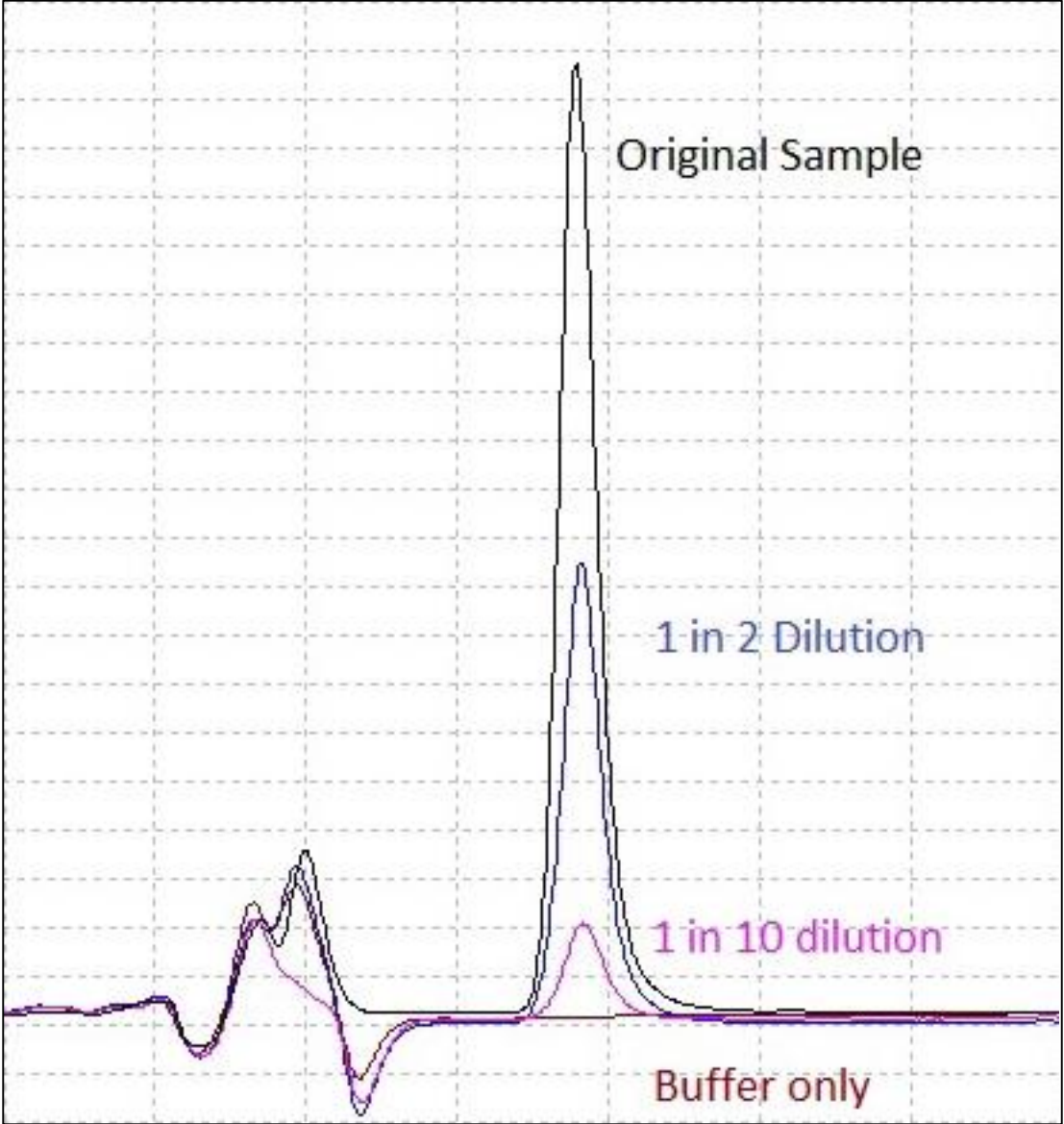

Supplement: Supplementary_materials.zip [file koni-06-07-1326437-s001.zip › Supplementary figure 1.pdf]

Supplementary Figure 2

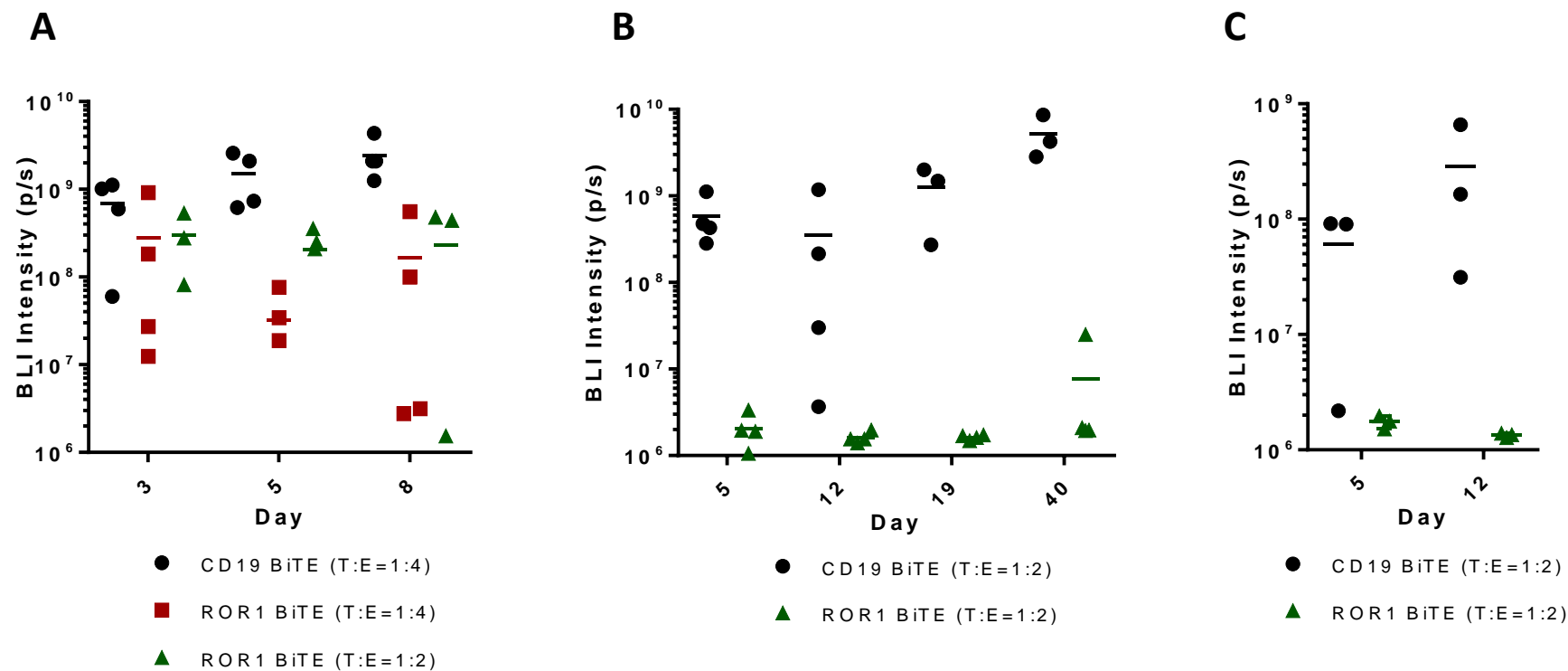

Supplement: Supplementary_materials.zip [file koni-06-07-1326437-s001.zip › Supplementary figure 2.pdf]

Supplementary Figure 3

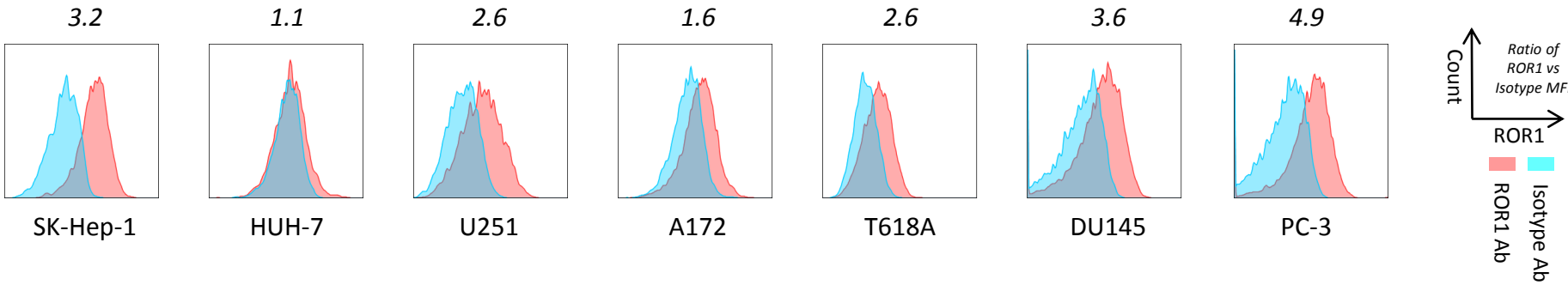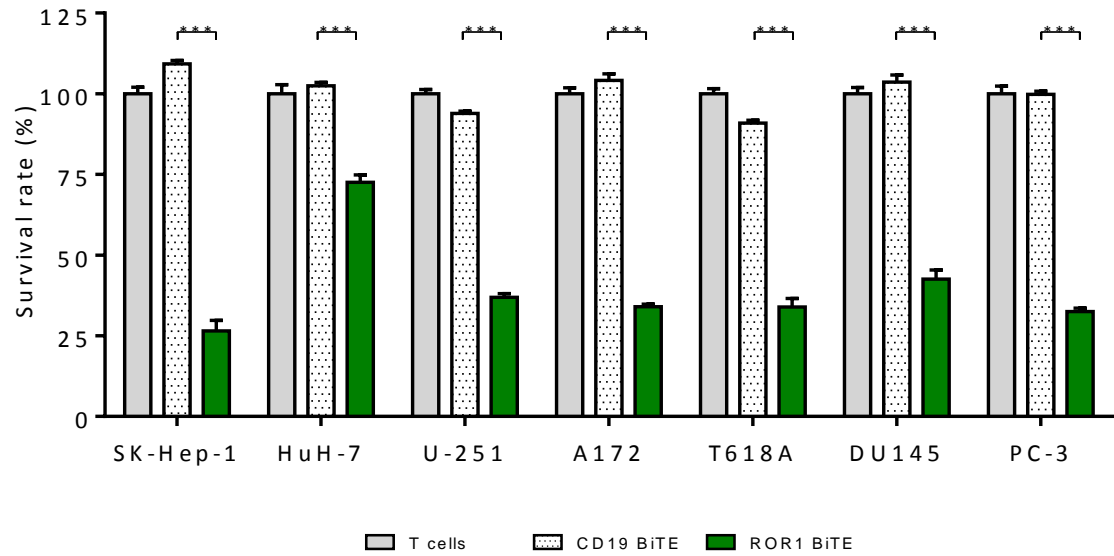

Supplement: Supplementary_materials.zip [file koni-06-07-1326437-s001.zip › Supplementary figure 3.pdf]
